# Self‐conscious emotions and breastfeeding support: A focused synthesis of UK qualitative research

**DOI:** 10.1111/mcn.13270

**Published:** 2021-10-15

**Authors:** Dawn Leeming, Joyce Marshall, Sophie Hinsliff

**Affiliations:** ^1^ Department of Psychology, School of Human & Health Sciences University of Huddersfield, Queensgate Huddersfield UK; ^2^ Division of Maternal Health, School of Human & Health Sciences University of Huddersfield, Queensgate Huddersfield UK

**Keywords:** breastfeeding, breastfeeding support, infant feeding, post‐natal care, qualitative methods, systematic review

## Abstract

Research on women's experiences of infant feeding and related moral discourse suggests that self‐conscious emotions may be highly relevant to breastfeeding support interactions. However, the emotional impact of receiving support has not been fully explored. The aim of this review is to re‐examine qualitative UK research on receiving breastfeeding support, in order to explore the role of self‐conscious emotions and related appraisals in interactions with professional and peer supporters. From 2007 to 2020, 34 studies met criteria for inclusion. Using template analysis to identify findings relevant to self‐conscious emotions, we focused on shame, guilt, embarrassment, humiliation and pride. Because of cultural aversion to direct discussion of self‐conscious emotions, the template also identified thoughts about self‐evaluation, perceptions of judgement and sense of exposure. Self‐conscious emotions were explicitly mentioned in 25 papers, and related concerns were noted in all papers. Through thematic synthesis, three themes were identified, which suggested that (i) breastfeeding ‘support’ could present challenges to mothering identity and hence to emotional well‐being; (ii) many women managed interactions in order to avoid or minimise uncomfortable self‐conscious emotions; and (iii) those providing support for breastfeeding could facilitate women's emotion work by validating their mothering, or undermine this by invalidation, contributing to feelings of embarrassment, guilt or humiliation. Those supporting breastfeeding need good emotional ‘antennae’ if they are to ensure they also support transition to motherhood. This is the first study explicitly examining self‐conscious emotions in breastfeeding support, and further research is needed to explore the emotional nuances of women's interactions with supporters.

**Key messages**

- Experiencing and managing self‐conscious emotions is part of infant feeding for many women, but this is underresearched.
- Engaging with breastfeeding support can challenge women during their transition to motherhood, evoking further self‐conscious emotions.
- Women may manage interactions with breastfeeding supporters carefully to minimise uncomfortable self‐conscious emotions and maintain a positive mothering identity, and this sometimes reduces the effectiveness of support.
- Breastfeeding supporters can significantly impact (and potentially undermine) women's development of a positive mothering identity. They need good emotional ‘antennae’, as self‐conscious emotions are often hidden.

## 1 INTRODUCTION

Those promoting breastfeeding have sometimes been accused of promoting guilt among women who formula feed (Taylor & Wallace, 2012). Therefore, it is perhaps unsurprising that research informing interventions to support breastfeeding has shied away from the troubling topics of guilt and shame. However, recent analyses suggest that negative self‐conscious emotions may play a significant part in many women's infant feeding experiences and that understanding such feelings may be important for developing effective support (e.g., Benoit et al., 2016; Smyth, 2018; Thomson et al., 2015). This systematic review re‐examines research on formal UK breastfeeding support to understand more fully the role of self‐conscious emotions in women's interactions with breastfeeding supporters such as midwives, health visitors, lactation consultants, trained breastfeeding support volunteers and other mothers attending organised peer breastfeeding support schemes. Many mother–infant pairs struggle initially to establish breastfeeding and benefit from skilled support (McFadden et al., 2017). Within the United Kingdom, assistance with breastfeeding can be delivered via groups, individual consultation or telephone, in varying contexts including postnatal wards, women's homes, clinic settings and informal drop‐ins offering specialised advice. Current guidelines on breastfeeding support recommend developing empathic relationships with mothers, which offer emotional support alongside information and technical advice on lactation and attachment at the breast (UNICEF UK, 2016a). However, despite these intentions, receiving support to overcome breastfeeding challenges may feel emotionally difficult to mothers who perceive the ‘support’ interaction as a situation that risks exposure of failure as a mother. Simply feeling ‘not normal’ as a woman or mother can trigger experiences of shame or embarrassment, particularly where there is a sense that others may discover this (Brown, 2007). Moreover, engagement with the gaze of health care workers can leave women feeling disapproved of and disempowered if they do not follow professionally approved feeding practices (Smyth, 2018). Therefore, interactions with those sanctioned as breastfeeding ‘experts’ may often be ripe for feelings of embarrassment, guilt, shame or even humiliation, rather than pride (Thomson et al., 2015).

### 1.1 Self‐conscious emotions

Although the range of self‐conscious emotions and the distinctions between them are debated (Gibson, 2019), their importance is widely agreed. Guilt, pride, shame, humiliation and embarrassment (or their avoidance) motivate achievement, shape moral and caring behaviour and promote action to repair relationships and social image, though negative self‐conscious emotions such as shame and guilt can be painful and debilitating (Tracy & Robins, 2007). According to Gilbert (2017), shame is particularly problematic. It is the emotional resonance of believing ourselves flawed and devalued in the eyes of actual or imagined others, damaging our sense of connection and leaving us paralysed and isolated. He contrasts this with guilt—a sense that we have *done* something wrong. With its narrower focus on specific actions or omissions, guilt may not leave us feeling as powerless or damaged as shame, though it can be difficult to tolerate if we cannot make reparation, and both emotions have been linked with mental health difficulties such as depression (e.g., Kim et al., 2011).

Both positive and negative self‐conscious emotions share common features—a focus on the self as the object of either our own or others' evaluation and a concern with how our actions or selves align with standards, rules or identity goals that are often based on shared cultural understandings (Lewis, 2016; Tracy & Robins, 2007). Although it may not always be possible to draw sharp distinctions between experiences labelled as different self‐conscious emotions (Gibson, 2019; Gilbert, 2003), these experiences can raise differing concerns. For example, the term guilt is usually invoked when there is a breach of moral standards, whereas embarrassment occurs when publicly breaching less consequential social rules (Gibson, 2019) and generally involves a sense of exposure (Lewis, 2016). Robins and Schriber (2009) note that experiences referred to as shame or pride usually involve success or failure. For shame, the sense of a failed or flawed self may relate either to moral standards or to other domains, for example, the body, stigmatising attributes, performance failures that impact identity or being mocked (Leeming & Boyle, 2013). Loss of status or rejection is seen as particularly important by some theorists for understanding shame and humiliation and considered less important for guilt and embarrassment (e.g., Gilbert, 2003; Scheff, 2003). These varying emotional experiences prompt different actions ranging from appeasement, reparation and apology, to avoidance, defensiveness and boastfulness. However, a common feature of these actions is that they facilitate complex social goals relating to identity concerns and management of the self in social life (Tracy & Robins, 2007). Varieties of self‐conscious emotion may therefore be expected in situations where we are aware of dominant cultural discourses about the kind of selves we ought to be and where measuring up to these moral standards or social conventions matters to us and, we assume, to those around us.

### 1.2 Self‐conscious emotions and motherhood

Motherhood—and more specifically infant feeding—might be one example. Mothering ideals may differ, but there is consensus that it is important to be a ‘good mother’ (Marshall et al., 2007). Women report that a sense of failure related to mothering is a common shame trigger (Brown, 2006), and several have argued that internalised, unrealistic expectations have made guilt an intrinsic part of motherhood (e.g., Liss et al., 2013). Contemporary notions of ‘good mothering’ can imply total focus on optimising the child's health, well‐being and development, regardless of cost to the mother (Head, 2017), with the implication that mothers have a *duty* to breastfeed (Woollard & Porter, 2017). Infant feeding practices are therefore shaped not only by social conventions but also by moral injunctions (Russell et al., 2021). This creates a ‘moral minefield’ that mothers must negotiate to demonstrate to themselves and others that they are a ‘good mother’ regardless of feeding method (Johnson et al., 2013; Ryan et al., 2010). Although this opens the possibility for pride in achievement, infant feeding often takes place within social environments which are *not* supportive of breastfeeding, despite its widely recognised health benefits (see Victora et al., 2016, for review). Breastfeeding may not fit easily with other demands of family life (Leeming et al., 2013) or local cultural contexts (Head, 2017). Women may feel embarrassed about public breastfeeding—as if they are disregarding the feelings of others (Boyer, 2018). On the other hand, some may feel shame or guilt about their perceived failure to establish breastfeeding (Williamson et al., 2012). These constraints on feeding choices are not always recognised and, instead, blame for using formula milk is internalised. It is not surprising that, in relation to breastfeeding, women may feel ‘shame if you do—shame if you don't’ (Thomson et al., 2015, p. 33). However, sustained investigation of self‐conscious emotions evoked by infant feeding is relatively recent, with just a few studies stating this as a primary aim, for example, exploring guilt in relation to public health messaging around breastfeeding (e.g., Benoit et al., 2016; Williams et al., 2012; Williams et al., 2013) or feeding method (Fallon et al., 2017; Komninou et al., 2017; Shepherd et al., 2017), or considering whether shame about perceived ‘failures’ with infant feeding may be mislabelled as guilt (e.g., Taylor & Wallace, 2012; Thomson et al., 2015) and a recent systematic review of research into shame and guilt in relation to feeding outcomes (Jackson et al., 2021).

### 1.3 Self‐conscious emotions in interaction with those supporting breastfeeding

Self‐conscious emotions often involve others (Gibson, 2019), so may be particularly likely when women discuss feeding their baby with those tasked to support their breastfeeding, potentially exposing perceived failures or successes and parts of the body deemed private. There is a sizeable body of research on women's experiences of receiving breastfeeding support, including several reviews (e.g., Joanna Briggs Institute, 2012; Schmied et al., 2011). However, to date, there has been limited empirical attention to self‐conscious emotional processes within breastfeeding support, though studies contributing to these reviews have suggested that women can feel judged or objectified by those assisting with breastfeeding.

Despite the social value of self‐conscious emotions, they can invoke tricky interpersonal dynamics (Sznycer, 2019). Therefore, understanding if and how self‐conscious emotions shape support interactions might help improve the effectiveness of support. Displays of pride may be socially risky (Wubben et al., 2012), and guilt can lead to social avoidance (Yu et al., 2017). However, shame and humiliation may be the most problematic, sometimes resulting in blaming, hostility and anger (Elison et al., 2014; Elshout et al., 2017), or withdrawal from and avoidance of others (e.g., Chao et al., 2011), though we may have limited awareness of, or deny, these processes (Brown, 2006; Scheff, 2003). Therefore, self‐conscious emotions are a slippery and taboo aspect of our lives to research (Scheff, 2016), which may influence relationships with others in ways that are problematic but unacknowledged. Lazare (1987) is one of the few to apply this to health care consultations. He argued that where clients or patients feel exposed, scrutinised or deficient, they may respond in ways that seem hostile or disrespectful, with the consequence of mutual blaming, labelling clients as ‘difficult’, and disengagement on both sides. This may be more likely where health care providers are sensitive to feeling shame themselves due to perceived criticism (Gilbert, 2017). With a few notable exceptions (e.g., Dolezal, 2015; Gibson, 2015, 2019; Gilbert, 2017; Thomson et al., 2015), the management of self‐conscious emotions in interactions with providers of health, social or maternity care has not been widely considered since Lazare's work. However, research outside the area of infant feeding suggests that others can play a role in helping individuals overcome problematic experiences of self‐conscious emotion through interpersonal processes such as meaningful connection, empathy, acceptance and affirmation (e.g., Brown, 2006; Leeming & Boyle, 2013).

### 1.4 Aim of paper

The aim of this paper is to re‐examine qualitative research reporting UK women's perspectives on receiving formalised breastfeeding support to understand whether and how self‐conscious emotions are relevant to interactions with those providing support. It is hoped that this will inform approaches to support that minimise difficult emotional experiences and complex interpersonal dynamics and support positive emotional responses to breastfeeding. As there is a recognised cultural aversion to direct discussion of self‐conscious emotions (Scheff, 2016), and such feelings can be fleeting or avoided, we have used a broad conceptualisation of self‐conscious emotions, including relevant appraisals. We focus on the ways in which women receiving breastfeeding support evaluate themselves and their actions, particularly where this appears to have emotional resonance. We also explore women's understanding of how those offering support have evaluated and treated them and their responses to this, including direct references to self‐conscious emotions and data providing insight into how women managed emotion and protected their sense of self during these encounters.

## 2 METHODS

### 2.1 Approach taken to synthesis

This was a focused thematic synthesis, exploring aspects of previous research on women's experiences of breastfeeding support, using a new conceptual framework. As in ‘framework‐based synthesis’ (Dixon‐Woods, 2011), we used a conceptually derived set of *a priori* themes for initial organisation and synthesis of findings but followed the methods of King and Brooks' (2017) ‘template analysis’. Therefore, unlike some forms of qualitative synthesis, we have not aimed to capture all aspects of receiving breastfeeding support interventions but to synthesise what this research reveals about self‐conscious emotions.

Although variants of self‐conscious emotions seem to be experienced universally, there are cultural differences in the issues about which people feel embarrassed, proud, guilty or ashamed, and the linguistic distinctions used to capture emotions (Gibson, 2019). We therefore limited the review to the United Kingdom, to enable better understanding of cultural context and of the support services researched.

### 2.2 Identifying relevant papers

Primary research studies were identified that reported women's experiences of interactive postnatal support to breastfeed a healthy term baby, from either health care professionals or organised peer support (e.g., breastfeeding groups or trained peer supporters). Breastfeeding support could be the main focus of the study or part of a broader investigation, such as experiences of general postnatal support or experiences of breastfeeding. Asynchronous online support via social media was not included, though telephone support was.

The search was conducted systematically using five electronic databases: CINAHL, Scopus, MEDLINE, PsychINFO and PubMed. Search terms included (Breastfeeding OR breast feeding OR breast‐feeding OR lactation OR breastfeed*) AND (support OR postnatal support OR post‐natal support OR lay support OR volunteer support OR social support OR breastfeeding counsellor OR breastfeeding education OR health education) AND (qualitative OR ethnography OR interviews OR phenomenology OR grounded theory OR thematic). Limits were applied for English language and to retrieve peer‐reviewed publications. We searched for UK studies from January 2007, following a rigorously conducted meta‐synthesis of women's experiences of breastfeeding support (Schmied et al., 2011), until 1 March 2020.

**Figure 1:**
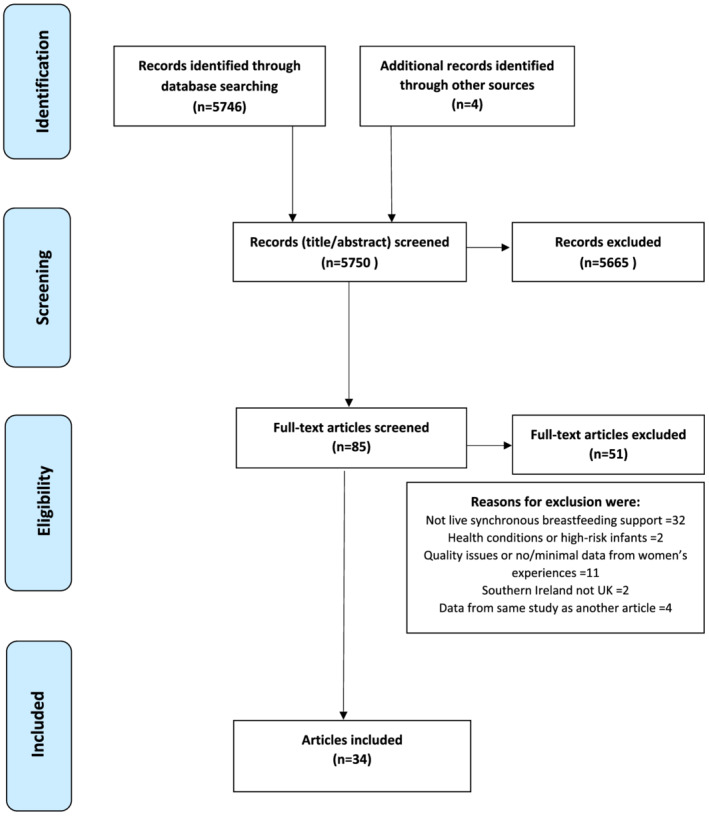
PRISMA flow diagram

Two researchers independently screened titles, abstracts and full‐text articles for potential inclusion. Any discrepancies were discussed with a third reviewer. Studies reporting experiences of breastfeeding support for specific demographic groups such as young mothers and ethnic minority groups were included, but studies on groups or settings with high risk of infant feeding difficulties were not (e.g., neonatal intensive care, illicit drug use, low birthweight and specific health conditions). Studies were excluded if they only reported health professionals' perspectives or analysed data from a previously published study. Quality appraisal of each paper was carried out by one of the research team, and reviewed by another team member, using a checklist devised for the study. This enabled assessment of each paper against the following criteria for inclusion: appropriate and clearly reported recruitment and data collection procedures; ethical approval; sufficient in‐depth qualitative data from mothers to provide insight into experiences of receiving breastfeeding support; and clear accounts of coding procedures, with sufficient quotes to justify and illustrate themes. As the aim was to reanalyse previous findings through a novel conceptual lens, studies were not rejected if the original analysis was mostly descriptive, with limited theoretical development, providing the data shed light on women's experiences of receiving support with breastfeeding and had been collected and reported in a rigorous and transparent manner. Due to the paucity of research on self‐conscious emotions when receiving support, and the often hidden nature of these experiences, it was important not to exclude otherwise trustworthy studies unnecessarily. Figure 1 summarises the review process—34 papers were included in the synthesis (see Table 1).

**Table 1:** Studies included in synthesis in reverse chronological order of year published

Authors	Year	Mothers providing data used in synthesis^a^	Nature of breastfeeding support	Methods used to gain mothers' accounts	Period since birth to data collection	Stated aim of study
Fraser et al.	2020	24 attending Children's Centre and initiating breastfeeding	Standard NHS maternity care,^c^ Children's Centres and breastfeeding support^d^ groups	Face‐to‐face interviews^e^	Up to 6 months	Examine mothers' experiences of breastfeeding support during first 6–8 weeks
Copeland et al.	2019	29 participating in novel breastfeeding peer support service developed for feasibility study	Individual peer support informed by the principles of Motivational Interviewing	Telephone interviews	Several weeks	Establish feasibility and acceptability of delivering novel intervention
Taylor et al.	2019	5 first‐time mothers initiating breastfeeding	Not specified. Recruited via standard NHS maternity care	Video diaries	Data collected prospectively after several days post‐natal	Explore how support impacted mothers' breastfeeding experiences during first few weeks
Thomson and Crossland	2019	302 accessing infant feeding peer support (hospital and community)	BFI‐accredited infant feeding individual and group peer support scheme (hospital and community)^f^	Open‐ended survey questions, plus telephone interviews with 8 of the participants	Not specified	Provide insights into how peer support can be operationalised to influence women's breastfeeding experiences. Informed by theoretical framework of behaviour change wheel
Edwards et al.	2018	18 first‐time mothers comprising 10 antenatal, regardless of feeding intentions, and 8 post‐natal, 7 of whom were breastfeeding	NHS midwife‐led maternity care clinic	Focus groups	11 days to 12 weeks	Explore women's and midwives' expectations, knowledge and experiences of breastfeeding initiation using social cognitive theory
Keevash et al.	2018	41 breastfeeding for any period of time	Not specified. Included standard NHS maternity care	Interviews (mode unspecified)	Up to 5 years	Understand breastfeeding experiences and what affects ability to continue breastfeeding
Hunt and Thomson	2017	13 breastfeeding for at least 5 days but *not* accessing breastfeeding peer support, though accessing other forms of support	NHS maternity care with BFI accreditation	Face‐to‐face interviews, telephone interviews and focus groups	Not specified	Explore reasons for non‐access to breastfeeding peer support (considered within context of experiencing other forms of support)
Jardine et al.	2017	10 first‐time mothers intending to breastfeed	Not specified. Recruited via standard NHS maternity care	Face‐to face interviews before birth and post‐natally	Approximately 4 weeks	Explore which psychosocial factors influence early discontinuation of breastfeeding, using theoretical domains framework
Ryan et al.	2017	49 breastfeeding within the past 2 years	Varied. Included standard NHS maternity care and voluntary sector breastfeeding support (e.g., La Leche League)	Face‐to‐face interviews	Not specified	Secondary analysis to understand role of agency in relation to breastfeeding initiation, maintenance and duration and breastfeeding support needs
Tan et al.	2017	9 attending breastfeeding cafés	BFI‐accredited breastfeeding cafés run by midwives and peer supporters trained in Solihull Approach^h^	Face‐to‐face interviews	Not specified	Explore women's perceptions of breastfeeding support café
Condon and Salmon	2015	15 gypsy, traveller or Roma mothers, regardless of feeding method	Standard NHS maternity care	Face‐to‐face interviews, some audio recorded	Up to 3 years	Explore mothers' and grandmothers' views on feeding in the first year of life, including support provided by health professionals
Fox et al.	2015	51 attending breastfeeding support ‘Baby Cafés’	Professional and peer support via Baby Cafés	Face‐to‐face interviews and focus groups	Varied	Examine experiences of breastfeeding and breastfeeding support in Baby Cafés
Hunter et al.	2015	15 aged 16–20 attending young parent groups in deprived areas who had considered breastfeeding	NHS post‐natal inpatient care	Focus groups and face‐to‐face interviews	Between 2 weeks and 21 months	Explore how inpatient experiences influenced feeding decisions and experiences, and breastfeeding support needs
Keely et al.	2015	28 defined as ‘obese’ who were no longer exclusively breastfeeding, despite intention to do so	Varied. Included standard NHS maternity care with some use of breastfeeding support clinics	Face‐to‐face interviews	6–10 weeks	Identify barriers to successful breastfeeding and reasons for introducing formula and/or stopping breastfeeding, and explore experiences of breastfeeding support services
Leeming et al.	2015	22 first‐time mothers initiating breastfeeding	Varied. Included standard NHS maternity care with breastfeeding ‘drop‐in’ and some use of voluntary sector support	2 face‐to‐face interviews and audio diaries	Audio diary: from 1 to 3 days. Interviews: 1–2 and 5–6 weeks	Explore how breastfeeding women experienced and made sense of their relationships with breastfeeding supporters
Spencer et al.	2015	22 breastfeeding for at least 11 days	Not specified. Accessed via standard NHS maternity care and included post‐natal support groups	Face‐to‐face phenomenological interviews	3–6 months	Explore experiences of breastfeeding women
Hinsliff‐Smith et al.	2014	22 first‐time mothers intending to breastfeed from area with lower rates of breastfeeding	NHS maternity care with BFI accreditation and some use of breastfeeding support groups	Face‐to‐face interviews and written diaries	Diaries: Up to 6 weeks. Interviews: unclear	Develop an understanding of experiences and challenges of breastfeeding in early post‐partum period
Lagan et al.	2014	78 using varied feeding methods from area with lower breastfeeding rates	NHS maternity care with BFI accreditation for hospital but not for community service	Focus groups and face‐to‐face interviews	4 to 8 months	Explore expectations and experiences of postnatal mothers in relation to infant feeding, and identify how care could be improved
Condon et al.	2013	6 pregnant teenagers and 23 teenage mothers aged 18 years or younger, regardless of feeding method	NHS maternity care with BFI accreditation	Focus groups and face‐to‐face interviews	Up to 2 years	Explore teenagers' experiences of breastfeeding promotion and support delivered by health professionals
Ingram	2013	163 receiving breastfeeding peer support service in areas with low breastfeeding rates	Individual peer support accredited by La Leche League	Online survey, with additional interviews for 14 mothers (13 telephone and 1 face‐to‐face)	2 weeks to 4 months	Mixed‐methods evaluation of peer support scheme, exploring effects of service on breastfeeding rates and perceptions of mothers, midwives and peer supporters
McFadden et al.	2013	23 of Bangladeshi origin breastfeeding in the past 5 years	Not specified. Included standard NHS maternity care	Face‐to‐face interviews	4 months to 5 years	Explore extent to which cultural context makes a difference to experiences of breastfeeding support for women of Bangladeshi origin
Thomson and Crossland	2013	885 using peer support telephone helplines	Breastfeeding peer support telephone helplines	Telephone interviews	Pregnancy to over 12 months post‐natal	Explore callers' experiences of help and support received via breastfeeding helplines
Guyer et al.	2012	6 middle‐class mothers breastfeeding in the past year	Not specified. Mentioned midwives and peer support group	Face‐to‐face phenomenological interviews	Up to 15 months	Increase understanding of experiences of breastfeeding mothers who are well educated and informed, but who struggle to meet WHO ideal of 6 months exclusive breastfeeding
Hoddinott, Craig, Britten, and McInnes	2012	36, mostly from disadvantaged areas, who intended to breastfeed or had breastfed a previous baby	Varied. Included standard NHS maternity care	2–8 serial face‐to‐face or telephone interviews, with individual women or in pairs with significant others	Late pregnancy to 6 months post‐natal	Investigate infant feeding experiences of women and their significant others from pregnancy until 6 months after birth to establish what would make a difference
Hoddinott, Craig, MacLennan, et al.	2012	372 from disadvantaged areas initiating breastfeeding and in contact with a telephone support service	Telephone support for breastfeeding women provided by post‐natal ward for 14 days following discharge, plus initial feeding support on ward	Open‐ended question in structured telephone interview, in‐depth face‐to‐face interview (40) and in‐depth follow‐up telephone interview (11)	Up to 8 weeks following discharge	Assess feasibility, acceptability and fidelity of feeding team intervention with an embedded RCT of team‐initiated and woman‐initiated telephone support after hospital discharge
Redshaw and Henderson	2012	Those mentioning infant feeding support when responding to free text questions about (i) post‐natal hospital care (*N* = 254) and (ii) overall maternity care (*N* = 196)	Standard NHS maternity care	National survey of women's experiences of maternity care	3 months	Use responses from broader survey to understand what is needed in early days to enable women to initiate and continue breastfeeding
Thomson, Crossland, and Dykes	2012	47 receiving breastfeeding peer support	Hospital and community breastfeeding peer support scheme (individual and group)	Face‐to‐face or telephone interviews	2 weeks to 17 months	Explore how breastfeeding peer support service facilitated hope
Thomson, Dykes, et al.	2012	26 from disadvantaged area engaging with breastfeeding peer support scheme	Community breastfeeding peer support scheme with incentives	Face‐to‐face or telephone interview	Mostly after 8 weeks when intervention finished	Investigate barriers and facilitators to incentive uptake, and meaning attributed to incentives
Brown and Lee	2011	33 exclusively breastfeeding for 6 months	Varied	Face‐to‐face or telephone interview	6–12 months	Explore attitudes and experiences of mothers
Thomson and Dykes	2011	15 prior to 12 months post‐partum, regardless of feeding method	NHS maternity care with BFI accreditation	Face‐to‐face interviews	4 weeks to 9 months	Provide a theoretical interpretation of ‘comprehensibility’, ‘manageability’ and 'meaningfulness' of women's experiences of infant feeding
Beake et al.	2010	20 on post‐natal wards, regardless of feeding intentions	NHS post‐natal inpatient care	Face‐to‐face interviews	A few days (still on ward)	Explore experiences and expectations of women receiving inpatient post‐natal care
Entwistle et al.	2010	7 low‐income mothers intending to breastfeed	Standard NHS maternity care with midwives receiving additional breastfeeding training	Face‐to‐face interviews	10–18 weeks	Explore views and experiences of low‐income women in relation to self‐efficacy and receiving breastfeeding support
Wade et al.	2009	16 receiving breastfeeding peer support	Breastfeeding peer support	2 focus groups	2 months to 3 years	Determine if breastfeeding peer supporters may offer benefits to breastfeeding women and their families other than increasing breastfeeding initiation and sustainability
Marshall et al.	2007	22 breastfeeding women—rural, suburban and inner city	Standard NHS maternity care	Face‐to‐face interviews	12 days to 6 months	Explore breastfeeding in the context of everyday living with a new baby and the support provided by midwives and health visitors

Abbreviations: BFI, Baby Friendly Initiative; NHS, National Health Service; RCT, randomised controlled trial; WHO, World Health Organization.

^a^ The review synthesised mothers' accounts of postnatal breastfeeding support. Several studies also included data from those providing support, other family members or observation (Condon & Salmon, 2015; Copeland et al., 2019; Edwards et al., 2018; Hoddinott, Craig, Britten, & McInnes, 2012; Hoddinott, Craig, MacLennan, et al., 2012; Hunt & Thomson, 2017; Ingram, 2013; Marshall et al., 2007; McFadden et al., 2013; Thomson & Crossland, 2019; Thomson, Dykes, et al., 2012). These data were not included in the synthesis nor were references to antenatal breastfeeding education or to postnatal support unrelated to infant feeding.

^b^ Originally established as Sure Start in the 1990s to support children and families in disadvantaged areas by providing services tailored to the local community.

^c^ Free care offered by UK National Health Service, which includes prenatal information about breastfeeding, post‐natal inpatient support from midwives to establish breastfeeding and later community support from health care staff with varied levels of training.

^d^ Within the United Kingdom, the terms ‘breastfeeding support’ and ‘infant feeding support’ can sometimes be used interchangeably because all services advocate breastfeeding where possible. Services labelled as the former may still offer support to women intending to breastfeed who switch to formula feeding.

^e^ Unless stated otherwise, ‘interviews’ were audio recorded and semistructured, with individual women.

^f^ Accredited as meeting best practice standards of UNICEF UK Baby Friendly Initiative.

^g^ ‘Breastfeeding café’ or ‘Baby Café’ denotes a breastfeeding support group run on a café‐style drop‐in basis, usually facilitated by health professionals or trained breastfeeding counsellors/lactation consultants and peer supporters.

^h^ An evidence‐based approach to early years parenting (Douglas, 2012) used in UK health visiting to promote emotional health and well‐being in infants and parents.

### 2.3 Data extraction

We were mindful of Scheff's (2016) argument that silence about shame and related emotions can extend to academic analyses, where the ‘s‐word’ may be ignored in favour of alternative interpretations of data. Therefore, our primary focus was original quotations and descriptive summaries of participants' accounts, though attention was also paid to authors' interpretations. For each paper, the findings section (or the parts of it related to support with infant feeding) was extracted for detailed coding in QSR's software package NVivo. We included findings on experiences related to support with formula feeding if support was from a service aimed primarily at encouraging breastfeeding, as is usually the case with infant feeding support within the United Kingdom. The remainder of the paper was examined (but not coded) to contextualise the data and findings.

### 2.4 Method of analysis and framework for examining self‐conscious emotions

Using template analysis (King & Brooks, 2017) enabled a focus on self‐conscious emotions and related appraisals by including tentative *a priori* themes within a coding template that was revised and expanded in response to the data. Template analysis was originally developed for thematic analysis of primary data though there are some examples of its use for qualitative synthesis (e.g., Au, 2007; Brown & Lan, 2013). Analysis of most papers fitted with King and Brooks' (2017) description of using template analysis within a contextualist framework, given the focus on interpretation of experience and interactions in particular contexts. This aligns with an understanding of self‐conscious emotions as interpretative and relational phenomena, comprising overlapping, loose clusters of embodied experiences with some similarities and also cultural variation (e.g., Gibson, 2019). Our use of terms such as ‘shame’, ‘guilt’ and ‘humiliation’ should therefore not be taken to imply that these are necessarily discrete, easily differentiated experiences.

**Figure 2:**
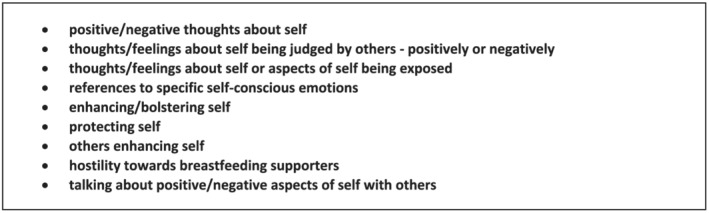
Initial *a priori* themes guiding early coding

**Figure 3:**
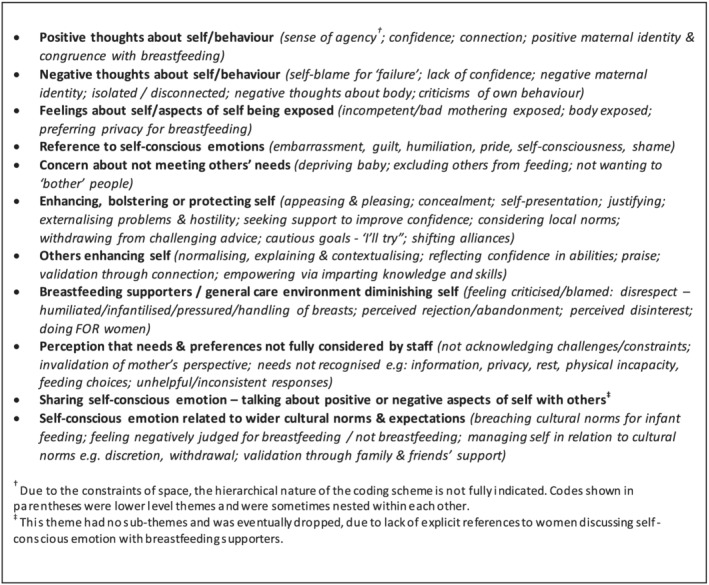
Intermediate overarching themes, indicating lower level codes within each

Drawing on the literature on self‐conscious emotions, an initial outline coding template was developed, which comprised several overarching *a priori* themes (see Figure 2). These were ‘soft’ *a priori* themes (King & Brooks, 2017), being broad, loosely defined and intended only to draw attention to key aspects of the data—self‐conscious emotions and self‐evaluative processes—rather than necessarily shaping the final organisation of the data. Additional lower level themes and new overarching themes were then developed inductively through line‐by‐line coding of six papers, until a fuller template was agreed, which was then used for coding a further 18 papers. This is shown in Figure 3 and includes minor revisions to themes that were made collaboratively during coding of these 24 papers.

**Figure 4:**
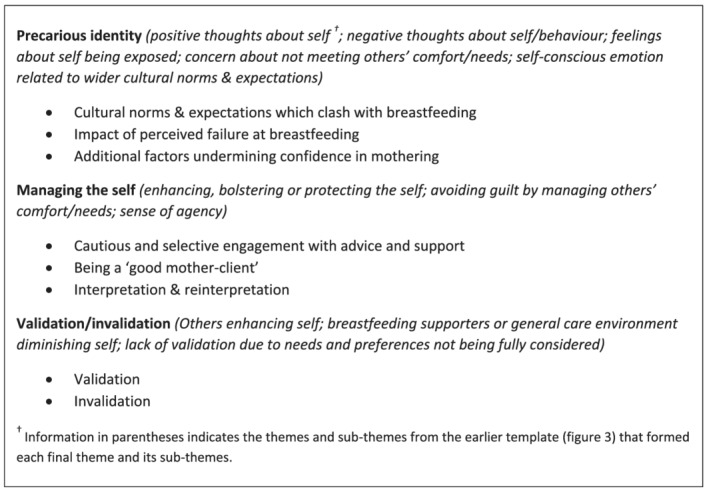
Final themes and subthemes

A more analytical and interpretative process of reviewing themes and data followed, where connections between themes and the context of studies were considered by the team. Some themes were decomposed, some relabelled and others reclustered, producing the final thematic structure (Figure 4), which was used for coding the remaining 10 papers. The themes were then audited and refined by one researcher rereading each of the 34 papers.

## 3 FINDINGS

The relevance of self‐conscious emotions to breastfeeding support was confirmed across the papers. Emotional aspects of receiving support were more apparent in studies that focused primarily on women's experiences, rather than evaluation of an intervention or care context. However, even if briefly, all papers included material relevant to our broad conceptualisation of self‐conscious emotions. Below, we begin with an overview of direct references to specific self‐conscious emotions. This is followed by discussion of the final themes from the template analysis (*precarious identity*, *managing the self* and *validation/invalidation*). These themes were developed through exploration of issues relevant to self‐conscious emotions, such as thoughts and feelings concerning self‐evaluation, exposure in front of breastfeeding supporters, perceived judgement by others involved in feeding support and management of emotional feelings in relation to these issues. Cultural aversion to direct discussion of many self‐conscious emotions means that these concerns are sometimes the only indicators of fleeting self‐conscious emotions or avoidance of these.

### 3.1 Direct references to specific self‐conscious emotions

Specific self‐conscious emotions were mentioned in relation to infant feeding in the findings of 25 of the 34 papers. Guilt was referred to most commonly (Beake et al., 2010; Brown & Lee, 2011; Edwards et al., 2018; Fox et al., 2015; Guyer et al., 2012; Hinsliff‐Smith et al., 2014; Hoddinott, Craig, Britten, & McInnes, 2012; Keely et al., 2015; Lagan et al., 2014; Marshall et al., 2007; Redshaw & Henderson, 2012; Spencer et al., 2015; Thomson & Crossland, 2013, 2019; Thomson & Dykes, 2011; Wade et al., 2009). Paradoxically, this could be guilt because of not successfully breastfeeding or because women *were* breastfeeding. Some also felt guilt about being a nuisance to staff. Several papers referred to embarrassment, related either to interactions with health care providers, which exposed their bodies or perceived shortcomings, or to the responses of others if they were to follow breastfeeding advice (Brown & Lee, 2011; Condon & Salmon, 2015; Condon et al., 2013; Edwards et al., 2018; Entwistle et al., 2010; Hinsliff‐Smith et al., 2014; Hoddinott, Craig, MacLennan, et al., 2012; Keevash et al.,  2018; Taylor et al., 2019; Thomson & Dykes, 2011). Although not often considered a specific self‐conscious emotion, several papers also referred to feelings of self‐consciousness in front of staff, visitors and family members, which seemed to have a similar meaning to embarrassment (Fox et al., 2015; Keely et al., 2015; Leeming et al., 2015; Ryan et al., 2017). References to pride were fewer, for example, when women felt they had succeeded in difficult circumstances (Brown & Lee, 2011; Condon & Salmon, 2015; Fox et al., 2015; Thomson & Dykes, 2011). Two papers referred to humiliating treatment, with women feeling patronised or unfairly criticised by staff (Redshaw & Henderson, 2012; Taylor et al., 2019). However, there were no direct references to women feeling shame. Even where self‐conscious emotions were not explicitly mentioned, there were indications that these emotions might be relevant to infant feeding, with references to failure, achievement, exposure, inadequacy or incompetence, self‐blame, feeling judged and references to letting others down, as discussed below.

### 3.2 Overview of themes from template analysis

Three overarching themes (*precarious identity*, *managing the self* and *validation/invalidation*), developed through the stages of the template analysis, suggested that obtaining support with breastfeeding involved interactions that could present multiple challenges to the self. Identity as a breastfeeding mother could be precarious, with the possibility of guilt, shame and embarrassment hovering on the periphery of many women's experiences, and more central for some. Many women appeared to engage in active management of their emotions while interacting with maternity care services, in order to maintain an intact self and avoid uncomfortable self‐conscious emotions. Those supporting breastfeeding could facilitate this emotion work where mothers perceived them as validating their mothering. Alternatively, ‘support’ could be experienced as invalidating and undermining.

### 3.3 Theme 1: Precarious identity as a breastfeeding mother

Participants in several studies struggled to navigate contradictions between the ‘breast is best’ message from those providing formal support, and alternative portrayals of breastfeeding as embarrassing, deviant or guiltily self‐indulgent from those around them. Others talked as if one could ‘succeed’ or ‘fail’ at breastfeeding, with a risk of being exposed as a shamefully inadequate mother. Confidence in breastfeeding could be further undermined by a sense of vulnerability and dependence. Although this theme focuses partly on the broader social context of women's breastfeeding experiences, outside of their interactions with services, it provides crucial context for understanding women's vulnerability to negative self‐conscious emotions when interacting with breastfeeding support services.

#### 3.3.1 Cultural norms and expectations that clash with breastfeeding

Encouragement to breastfeed could be counter to local cultural norms or social conventions (Condon et al., 2013; Entwistle et al., 2010; Fox et al., 2015; Marshall et al., 2007; Ryan et al., 2017; Tan et al., 2017). A study of young mothers provided an example:

1Some feared that to choose to breastfeed would transgress their own social norms:

*Midwives and doctors they try and persuade you to do it* [breastfeed] *because it is classed as a normal thing … but I don't know* (Condon et al., 2013).

A sense that breastfeeding was deviant could be exacerbated if women had rarely seen others breastfeed (Condon et al., 2013; Hunt & Thomson, 2017; Tan et al., 2017; Thomson & Dykes, 2011). Many participants noted concerns about disapproval or embarrassment if breastfeeding might be observed by others (Condon & Salmon, 2015; Entwistle et al., 2010; Fox et al., 2015; Hinsliff‐Smith et al., 2014; Keely et al., 2015; McFadden et al., 2013; Thomson & Dykes, 2011), particularly if they were overweight (Keely et al., 2015), inexperienced (Leeming et al., 2015; Ryan et al., 2017) or feeding an older child (Keevash et al., 2018).

Lack of familiarity could reduce confidence establishing breastfeeding, particularly for some groups, such as those on low incomes or younger mothers (Entwistle et al., 2010; Fox et al., 2015). Breastfeeding could also feel morally dilemmatic if women felt it interfered with the expectations of a ‘good’ mother or partner, such as caring for other children (Hoddinott, Craig, Britten, & McInnes, 2012; Hunt & Thomson, 2017; Spencer et al., 2015), maintaining a sexual relationship (Marshall et al., 2007), allowing others to participate in bottle‐feeding (Guyer et al., 2012; Hoddinott, Craig, Britten, & McInnes, 2012; Marshall et al., 2007; McFadden et al., 2013; Spencer et al., 2015; Thomson & Dykes, 2011) or meeting the baby's perceived nutritional and other needs (Brown & Lee, 2011; Fox et al., 2015; Guyer et al., 2012; Hoddinott, Craig, Britten, & McInnes, 2012; Keely et al., 2015; Marshall et al., 2007; Spencer et al., 2015):

It took four or five weeks for her to regain her birth weight … and you kind of feel really guilty, don't you? Like maybe I'm making the wrong choice here—maybe I'm being selfish by not giving her a bottle (Fox et al., 2015).

At times, these concerns, which were at odds with the advice from infant feeding advisors, were prompted by comments from sceptical family members and friends:

I have had many comments such as ‘you're not still breastfeeding are you?’ ‘Surely she's going to starve on just milk’ (Brown & Lee, 2011; see also Entwistle et al., 2010; Fox et al., 2015; Keely et al., 2015; Spencer et al., 2015).

Some reported family and friends implying they were foolish to make life difficult for themselves and others by continuing to breastfeed (Brown & Lee, 2011; Entwistle et al., 2010; Fox et al., 2015; Keely et al., 2015; Spencer et al., 2015).

Where studies reported a clash between the advice offered by breastfeeding supporters and local expectations of ‘good’, sensible mothers, this did not necessarily mean women stopped breastfeeding, but it could become emotionally difficult and isolating (Tan et al., 2017). Participants and researchers often referred to this sense that breastfeeding constrained ideal motherhood as ‘guilt’. However, some participants explained how these perceived failures undermined their mothering identity and expressed concerns about how they appeared to others, which suggested that shame or embarrassment may also have been relevant.

Some studies presented an alternative picture, where at least some members of a mother's immediate network supported her breastfeeding (Brown & Lee, 2011; Condon & Salmon, 2015; Entwistle et al., 2010; Fox et al., 2015; Hoddinott, Craig, Britten, & McInnes, 2012; Marshall et al., 2007). For these women, identity as a breastfeeding mother seemed less precarious, and this support helped to resist negativity from elsewhere about their choice to breastfeed and engage with breastfeeding support.

#### 3.3.2 Impact of perceived ‘failure’ at breastfeeding

Despite the potential for mothers to feel guilty about the impact of breastfeeding on others, there were more references to guilt and self‐blame about *not* breastfeeding or perceived breastfeeding failures (Edwards et al., 2018; Fox et al., 2015; Guyer et al., 2012; Hinsliff‐Smith et al., 2014; Hoddinott, Craig, Britten, & McInnes, 2012; Hunt & Thomson, 2017; Hunter et al., 2015; Jardine et al., 2017; Keely et al., 2015; Keevash et al., 2018; Lagan et al., 2014; Marshall et al., 2007; Redshaw & Henderson, 2012; Spencer et al., 2015; Thomson & Crossland, 2013; Thomson & Dykes, 2011; Wade et al., 2009). A few studies noted particularly high levels of distress about ‘failures’ (Guyer et al., 2012; Jardine et al., 2017; Keely et al., 2015; Keevash et al., 2018; Redshaw & Henderson, 2012; Thomson & Dykes, 2011), for example:

… I cried most of the night on day three, and again I think it was guilt as well, except that I didn't really want to stop. I kept saying ‘I'll just keep trying. I'll wait another day and see what happens’ (Keely et al., 2015).

The term commonly used to describe emotional responses to these perceived failures was ‘guilt’, though women in several studies seemed to be referring to a sense of inadequacy or failure as a mother, or even as a woman (Fox et al., 2015; Hinsliff‐Smith et al., 2014; Hunt & Thomson, 2017; Hunter et al., 2015; Jardine et al., 2017; Keevash et al., 2018; Lagan et al., 2014; Leeming et al., 2015; Redshaw & Henderson, 2012; Spencer et al., 2015; Tan et al., 2017; Thomson & Dykes, 2011), for example, concluding, ‘there's something wrong with me’ (Hunt & Thomson, 2017). Redshaw and Henderson (2012) noted this could leave women feeling ‘like a “*bad person*” or “*a failure*” who “*had let the baby down*” ’ and quoted one participant adding ‘my baby didn't take to breastfeeding and I felt humiliated’. One of Keevash et al.'s (2018) participants said, ‘I felt it [breastfeeding] was all tied up in my worth as a mother’.

Several authors linked distress and feelings of self‐blame or inadequacy to a ‘rules‐based’ approach, which implied there was only one way to breastfeed (Hoddinott, Craig, Britten, & McInnes, 2012; Hunt & Thomson, 2017) or prior expectations that breastfeeding would be straightforward or instinctively ‘natural’ (Fox et al., 2015; Fraser et al., 2020; Hinsliff‐Smith et al., 2014; Hunt & Thomson, 2017; Hunter et al., 2015; Keevash et al., 2018; Lagan et al., 2014; Marshall et al., 2007; Ryan et al., 2017; Thomson & Dykes, 2011). Interaction with skilled helpers could then risk exposing an ‘inadequate’ self who did not measure up as a breastfeeding mother:

I didn't want my baby screaming if nobody else's baby was screaming [on post‐natal ward] … and didn't want the nurses coming in all the time or the midwives thinking what's wrong with her (.) She's not managing very well (Leeming et al., 2015).

Other studies also suggested a sense of surveillance and expectations of negative judgement from health care professionals and peer supporters (Guyer et al., 2012; Hoddinott, Craig, Britten, & McInnes, 2012; Hunt & Thomson, 2017; Hunter et al., 2015; Keely et al., 2015; Keevash et al., 2018; Lagan et al., 2014; Marshall et al., 2007; Redshaw & Henderson, 2012; Spencer et al., 2015; Taylor et al., 2019; Thomson & Crossland, 2013; Thomson & Dykes, 2011).

#### 3.3.3 Additional factors undermining confidence in mothering

Women's confidence as mothers, and therefore presumably their interactions with health care providers, was also undermined by feeling exhausted or vulnerable after childbirth (Edwards et al., 2018; Hunt & Thomson, 2017; McFadden et al., 2013; Redshaw & Henderson, 2012; Ryan et al., 2017), recovering from a caesarean section (Entwistle et al., 2010; Keely et al., 2015), lack of experience with and knowledge about babies (Beake et al., 2010; Leeming et al., 2015; Ryan et al., 2017), unfamiliarity with hospital routines and environments (Beake et al., 2010; Entwistle et al., 2010; Ryan et al., 2017), isolation (Tan et al., 2017) and concerns about body size (Keely et al., 2015). There was a recurrent theme of women lacking confidence in their ability to breastfeed (see particularly Entwistle et al., 2010; Leeming et al., 2015; Marshall et al., 2007).

#### 3.3.4 Concluding comments on precarious identity as a breastfeeding mother

Infant feeding could present considerable challenges to the task of becoming a ‘good’ mother, creating guilt, embarrassment or a sense of shameful inadequacy. However, several studies also reported breastfeeding contributing to feelings of bonding, pride and achievement, even where there were initial difficulties (Brown & Lee, 2011; Entwistle et al., 2010; Fox et al., 2015; Guyer et al., 2012; Jardine et al., 2017; Lagan et al., 2014; Leeming et al., 2015; Marshall et al., 2007; Thomson & Crossland, 2013; Thomson & Dykes, 2011). For example:

I said to my husband if anyone ever asks me what my greatest achievement is, this is it, never mind getting a degree or anything like that, this is my greatest achievement, feeding my baby through all that shit (Thomson & Dykes, 2011).

However, there was some ambivalence as pride could be seen as ‘silly’ (Fox et al., 2015) or inappropriate because breastfeeding was ‘normal’ (Brown & Lee, 2011).

This theme suggests that although breastfeeding can be an important part of developing a positive mothering identity, this can be precarious. For many women, it was not always clear how to be a ‘good’ mother with regard to feeding their baby, because breastfeeding challenged their moral and cultural context. The additional challenges of new parenthood, including childbirth itself, further undermined their ability to navigate these contradictory pressures and could place them in an emotionally precarious position when engaging with breastfeeding support.

### 3.4 Theme 2: Managing the self in interaction with breastfeeding supporters

These conclusions do not necessarily mean that women across the studies experienced strong negative self‐conscious emotions, though some did. Many enjoyed breastfeeding and felt well supported. Several studies also found women actively engaging in what has been called ‘emotion work’ (Hochschild, 1979) or ‘moral work’ (Ryan et al., 2010) to resist negative self‐conscious emotions, promote positive emotions and preserve a positive view of their selves and their mothering. However, as discussed below, sometimes this interfered with effective use of breastfeeding support.

#### 3.4.1 Cautious and selective engagement with advice and support

Engagement with breastfeeding support could be an important strategy for repairing or avoiding feelings of failure, guilt or inadequacy:

I was tired and frustrated and feeling guilty that my baby always seemed to be unsatisfied after her feeds … She [peer supporter] visited me two more times … until I had a complete turnaround and felt like I was succeeding (Thomson & Crossland, 2019).

However, as Ryan et al. (2017) noted, some women were cautious and selective about support and advice, and this seemed particularly the case where they perceived that following advice to the letter might lead to guilt or embarrassment. For example, women discussed modifying advice by adding bottles of formula, expressing extensively, introducing solids early or stopping breastfeeding. This could avoid guilt about breastfeeding competing with the perceived needs of the baby or others, or avoid embarrassment about public feeding (e.g., Condon et al., 2013; Hoddinott, Craig, Britten, & McInnes, 2012; Hunt & Thomson, 2017; Marshall et al., 2007; Spencer et al., 2015). However, these modifications were not compatible with exclusive breastfeeding. Sometimes infant feeding advisors supported these attempts to balance different pressures, but where women thought that supporters might disagree, they sometimes withdrew or avoided discussion (Hoddinott, Craig, Britten, & McInnes, 2012; Spencer et al., 2015; Taylor et al., 2019). Some mothers spoke of reluctance to engage with additional support, such as peer support, for fear they would not ‘measure up’ (Fraser et al., 2020; Hunt & Thomson, 2017; Keely et al., 2015; Ryan et al., 2017; Spencer et al., 2015; Taylor et al., 2019; Thomson & Dykes, 2011). Hunt and Thomson (2017) commented on this avoidance of potentially shaming exposure:

The conflict between women's self‐perceptions of being ‘a failure’ doing ‘a crappy job’ and the imagined ‘successful’ breast‐feeders who accessed the [peer‐support] groups was a key barrier to access.

However, for other women, contact with other breastfeeding women could be a strategy for *strengthening* identity and confidence as breastfeeding mothers (see Section 3.5.1). This could involve shifting alliances as they avoided previous friends and made new friends, for example, via Baby Cafés:

I thought I need to find a place to go to where I've got likeminded people and that grew my confidence to be honest as a new mum and as a breastfeeding mum (Fox et al., 2015).

#### 3.4.2 Being a ‘good mother–client’

Concerns about impression management included behaving appropriately as a client of maternity services. Some mothers ‘felt guilty about bothering staff’ (Beake et al., 2010), particularly on busy postnatal wards (Beake et al., 2010; Hoddinott, Craig, Britten, & McInnes, 2012; Hunter et al., 2015; Redshaw & Henderson, 2012; Ryan et al., 2017) and also in community services (Hoddinott, Craig, MacLennan, et al., 2012; Thomson & Crossland, 2013). They avoided pressing the buzzer, or asking for help, to avoid the guilt or embarrassment of becoming a ‘nuisance’. However, this reduced the likelihood of effective support. In one study, a younger mother explained the creative strategies she had employed:

Sometimes I used to press the buzzer, sort of put it back, and they'd come and I'd say ‘oh, oh, I must have leant on it!’ And then I'd say ‘oh, while you're here … ’, because I just felt like I was being such a nuisance (Hunter et al., 2015).

Spencer et al. (2015) noted other forms of subterfuge where women concealed their nonadoption of health care professionals' advice, or their difficulty following it. They called this ‘Illusions of compliance’, and examples are also noted by Hoddinott, Craig, Britten, and McInnes (2012), Taylor et al. (2019) and Thomson and Dykes (2011). Concealment of difficulties, or even hiding bottles, not only avoided battles with health care professionals or criticism but also avoided exposing what some women saw as an inability to cope:

I don't know if it's that you feel like you're being checked up on, so you know, you don't wanna say oh I'm struggling, because you're kinda passing a test, you feel that you're competent or not … (Spencer et al., 2015).

A stated intention to ‘try’ breastfeeding was noted by Condon et al. (2013) and Spencer et al. (2015) as another way of being a good mother–client, enabling women to claim good intentions, while leaving an exit route open, ‘rather than make a choice to formula feed from the start’ (Spencer et al., 2015).

Some strategies for managing emotion by being a good client were more straightforward and involved seeking ‘approval’ for infant feeding decisions directly from health care professionals or avoiding actions they assumed were not sanctioned (Entwistle et al., 2010; Guyer et al., 2012; Hunter et al., 2015; Lagan et al., 2014; Leeming et al., 2015; Spencer et al., 2015).

#### 3.4.3 Interpretation and reinterpretation

Emotion work also included reinterpreting events, which challenged maternal identity. As discussed under Section 3.5.1, breastfeeding supporters sometimes facilitated this, though positive interpretation could be an internally driven process, focusing on achievements or adopting a ‘can do’ approach (Brown & Lee, 2011; Entwistle et al., 2010; Jardine et al., 2017; Keely et al., 2015; Ryan et al., 2017):

I am able to do it [breastfeed], I know that I'm able to do it, as soon as you turn around and say you can't do something, then you instantly—you're not gonna do it (Jardine et al., 2017).

However, interpretations that preserved a positive sense of self could be at odds with continuation of breastfeeding. Sometimes women justified formula feeding with reference to pain, struggles, inadequate milk supply, the baby's well‐being or family relationships:

… don't really regret stopping [breastfeeding] because it was quite stressful for the both of us so and he's happier now, very happy and chubby (Keevash et al., 2018; see also Hoddinott, Craig, Britten, & McInnes, 2012; Marshall et al., 2007; Redshaw & Henderson, 2012).

Some justifications for discontinuing breastfeeding also preserved self‐esteem by attributing this to lack of support from health care professionals and limited or misleading information (Beake et al., 2010; Fox et al., 2015; Keely et al., 2015; Redshaw & Henderson, 2012). Whether or not these accounts are ‘true’, they indicate the pressure felt by women to account for their infant feeding decisions and direct blame somewhere. Hoddinott, Craig, Britten, and McInnes (2012) noted that some families expressed doubts about the improved health outcomes of breast milk compared with formula milk, concluding that this reinterpretation served to ‘to counter any implications of being a ‘bad mother’ or putting a child at risk’. Criticisms of health care advice and staff were only expressed as overt anger in one of the two diary studies (Taylor et al., 2019). Nonetheless, for a few women, this interpretative emotion work led to entrenched positions where breastfeeding became ‘unrealistic’, formula feeding became ‘reasonable’ and those advocating breastfeeding were to be resisted, avoided or blamed.

#### 3.4.4 Concluding comments on managing the self

Engagement with breastfeeding support was not a straightforward process for many women as it could mean far more to them than simply receiving infant feeding advice and guidance. For many, their mothering identity was at stake and interactions needed careful management to maintain emotional well‐being.

### 3.5 Theme 3: Validation/invalidation

Interactions with peer supporters and health care professionals could support emotion work by positively affirming women's mothering and countering self‐doubt, guilt and shame. However, many women indicated that care environments had exacerbated negative feelings. Below, we discuss this by considering whether women felt validated or invalidated by those offering support and guidance. The term ‘validation’ does not necessarily mean that a woman felt supporters agreed with her infant feeding decisions but that she thought they respected and accepted her and perceived her decisions to be meaningful and understandable given her circumstances. We use the term ‘invalidation’ to capture not just an absence of validation but a sense from women's accounts that others devalued and disrespected them, were disinterested or critical, and undermined their identity as a mother.

#### 3.5.1 Validation

Most papers reported some participants saying breastfeeding support had helped them to feel better about their mothering, through two overlapping processes of validation: (i) reflecting back a positive view of the woman and her mothering and (ii) meaningful connection and acceptance.

##### Positive feedback

This first form of validation could promote feelings of capability, achievement or pride and help to counter potential shame, guilt or embarrassment. Postive feedback could be simple reassurance that feeding was successful and the baby gaining weight (e.g., Taylor et al., 2019; Thomson & Crossland, 2019; Thomson, Crossland, & Dykes, 2012). Women who lacked confidence also valued a ‘you can do’ attitude from professional and peer supporters, both in the early days of breastfeeding and later when tackling issues such as public feeding or working (e.g., Brown & Lee, 2011; Fox et al., 2015; Ingram, 2013; Keely et al., 2015; Keevash et al., 2018; Leeming et al., 2015; Marshall et al., 2007; Redshaw & Henderson, 2012; Tan et al., 2017; Thomson & Crossland, 2013, 2019; Thomson, Crossland, & Dykes, 2012; Thomson & Dykes, 2011). For example, referring to a breastfeeding clinic, one woman said:

I would be like, ‘I can't do it, I can't do it’ and I would go back thinking ‘I'm going to tell them I can't do it’ and they would make me feel better again … (Keely et al., 2015).

Affirmation of women's ability to breastfeed could validate them more generally as mothers:

… they [peer supporters] tell me, you're doing a great job and it just makes you feel great. Oh I'm being a good mum, I'm being a great mum, I'm doing it right, it's nice (Thomson, Crossland, & Dykes, 2012).

Health care professionals could be particularly helpful in countering negative self‐belief. They could offer reassurance that breastfeeding difficulties were not unusual, were surmountable, could be temporary and were not indicative of deficient or incompetent mothering (Condon et al., 2013; Hinsliff‐Smith et al., 2014; Hoddinott, Craig, Britten, & McInnes, 2012; Hoddinott, Craig, MacLennan, et al., 2012; Ingram, 2013; Leeming et al., 2015; Marshall et al., 2007; Tan et al., 2017; Taylor et al., 2019). Peer supporters could also contextualise, reinterpret and normalise difficulties, countering a sense of failure:

She made me feel that I was not the only person in the world having this problem, I failed to feed my daughter and … thought breastfeeding was natural, I thought it must be me. So having someone explain it is normal and not completely easy for everyone (Thomson & Crossland, 2013; see also Brown & Lee, 2011; Copeland et al., 2019; Fox et al., 2015; Tan et al., 2017; Thomson & Crossland, 2019; Thomson, Crossland, & Dykes, 2012).

Peer support groups could play an additional role in reflecting a positive view of breastfeeding, by constructing an environment where ‘breastfeeding was the norm’ (Tan et al., 2017; see also Copeland et al., 2019) and where breastfeeding was valued and shared, including the challenges (see also Brown & Lee, 2011; Fox et al., 2015; Fraser et al., 2020; Guyer et al., 2012; Thomson, Crossland, & Dykes, 2012; Thomson & Dykes, 2011). Therefore, although peer contact required women to risk feeling that they did not measure up (see Section 3.4.1), it could be an important means of normalising and validating breastfeeding and resisting feelings of deviance or embarrassment.

##### Connection and acceptance

Positive experiences of peer support also supported women's emotion work by providing validation through connection with and acceptance by others—the opposite experience to shame. Most studies noted the importance of peer or professional supporters having a warm, patient, kind, respectful or caring manner. Some studies highlighted what Hoddinott, Craig, Britten, and McInnes (2012) call a ‘woman‐centred’ approach, which does not focus solely on maintaining breastfeeding but also shows that women are valued, by considering their individual situations and needs. Women appreciated breastfeeding supporters trying to understand their perspectives, concerns and experiences in a non‐judgemental manner, which respected their choices (Entwistle et al., 2010; Fox et al., 2015; Ingram, 2013; Tan et al., 2017; Taylor et al., 2019; Thomson & Crossland, 2013, 2019; Thomson, Dykes, et al., 2012) and promoted agency (Ryan et al., 2017). This not only helped to bolster positive self‐appraisals and related emotional experiences but could also enable disclosure of concerns about taboo topics (Ingram, 2013; Tan et al., 2017; Thomson, Dykes, et al., 2012), which allows supporters to challenge any negative self‐beliefs—the core of negative self‐conscious emotions. Where supporters accepted feeding practices that did not meet breastfeeding ideals but enabled some breast milk to be offered, this could help counter a sense of guilt or failure and acknowledge a woman's efforts in challenging circumstances (Hoddinott, Craig, Britten, & McInnes, 2012; Thomson & Crossland, 2013; Thomson, Crossland, & Dykes, 2012; Wade et al., 2009). This woman‐centred approach left mothers feeling that *they* and their wider concerns mattered, rather than just their ability to feed their baby optimally, though Entwistle et al. (2010) note the potential of this approach to undermine breastfeeding. Women valued other behaviours that indicated they mattered to supporters, such as spending time with them, being readily available, initiating contact and actively listening (Beake et al., 2010; Copeland et al., 2019; Hoddinott, Craig, Britten, & McInnes, 2012; Hunter et al., 2015; Ingram, 2013; Ryan et al., 2017; Tan et al., 2017; Thomson & Crossland, 2013, 2019; Thomson, Crossland, & Dykes, 2012; Thomson, Dykes, et al., 2012). Thomson, Dykes, et al. (2012) found that women receiving individually tailored gifts as incentives for breastfeeding interpreted these as a sign that they mattered as a person rather than a feeding agent. The gifts were ‘connectors’, communicating that the peer support scheme ‘cared about’ the women.

#### 3.5.2 Invalidation

Despite the importance of validation, many papers reported accounts from at least one or two women of interactions that could lead to shame, embarrassment or humiliation through women being demeaned or disregarded by those who were meant to support their breastfeeding. Redshaw and Henderson (2012) summarised several participants' portrayal of negative staff attitudes when supporting infant feeding on postnatal wards:

Negative staff attitudes or behavior ranged from women feeling ‘bullied’ or ‘judged’, being ‘shouted at’, midwives acting in a ‘condescending manner’, being ‘insensitive’, ‘inconsiderate’, ‘disrespectful’, and ‘rude’.

Similar experiences were reported elsewhere (Beake et al., 2010; Fox et al., 2015; Hinsliff‐Smith et al., 2014; Hoddinott, Craig, Britten, & McInnes, 2012; Hunter et al., 2015; Ryan et al., 2017; Taylor et al., 2019; Thomson & Crossland, 2013) and were described as humiliating (Taylor et al., 2019). Several women complained of midwives and others ‘manhandling’ or ‘grabbing’ their breasts (Edwards et al., 2018; Guyer et al., 2012; Hunter et al., 2015; Marshall et al., 2007; Redshaw & Henderson, 2012; Ryan et al., 2017; Taylor et al., 2019; Thomson & Dykes, 2011). They found this disrespectful and an embarrassing invasion of privacy:

… but it's a stranger in your home you don't know, grabbing your breasts, trying to shape them in a certain way to get it into your baby's mouth … that is the experience I had in hospital and at home. It's bloody awful, really embarrassing, I could actually cry (starting to cry) (Thomson & Dykes, 2011).

Women also complained of being disrespected as autonomous adults through pressure to make infant feeding decisions, dismissal of their ideas, judgemental surveillance or infantilisation (Condon et al., 2013; Fox et al., 2015; Guyer et al., 2012; Hinsliff‐Smith et al., 2014; Hoddinott, Craig, Britten, & McInnes, 2012; Hunt & Thomson, 2017; Hunter et al., 2015; Keevash et al., 2018; Lagan et al., 2014; Leeming et al., 2015; Marshall et al., 2007; Redshaw & Henderson, 2012; Ryan et al., 2017; Spencer et al., 2015; Taylor et al., 2019; Thomson & Crossland, 2013, 2019; Thomson & Dykes, 2011). Such experiences could ‘*make you doubt* yourself’ (Thomson & Crossland, 2019) and therefore might be ripe for feelings of embarrassment, guilt, shame or humiliation, though emotional responses were often implied or left unstated in interview data. Drawing on detailed video diaries, rather than the interviews used in most studies, Taylor et al. (2019) noted ‘deep emotional turmoil’ and anger in response to feeling ‘scrutinised and humiliated’ by staff and suggested that the extent of these feelings would have been difficult to capture in an interview.

Some women described more subtle forms of invalidation where they felt uncared for because staff seemed disinterested in their needs or unavailable (Fraser et al., 2020; Ryan et al., 2017; Taylor et al., 2019) or they felt disrespected because their needs and preferences were not acknowledged, for example, with regard to breastfeeding support, privacy or rest (Beake et al., 2010; Hunter et al., 2015; Keely et al., 2015; Redshaw & Henderson, 2012; Ryan et al., 2017). Although these various forms of invalidation were experienced within community and peer support services, they were more frequently discussed in relation to hospital care. For example, Hunter et al. (2015) referred to:

Task‐based, routinised care, during which the young women were treated as objects rather than self‐determining individuals … (see also Taylor et al., 2019).

#### 3.5.3 Concluding comments on validation–invalidation

The first two themes illustrated how some women approached motherhood and infant feeding feeling under surveillance and expecting to be judged by maternity care workers, particularly if not breastfeeding. Taylor et al. (2019) linked this to a medicalised, risk averse and authoritarian approach to infant feeding—checking if mothers and babies were on the ‘right track’. Thus, prior expectations of scrutiny may have amplified any subtle signals of disrespect, pressure or blame from staff, and as Redshaw and Henderson (2012) note, ‘a single unpleasant incident sometimes marred postnatal care’. As identity was already precarious for some, and negative self‐appraisals evident, they did not need to experience active *in*validation in order to struggle with an absence of validation and reassurance, and the emotional consequences of this.

## 4 DISCUSSION

This was a focused synthesis, exploring self‐conscious emotions, and was not an attempt to capture all aspects of women's experiences of receiving breastfeeding support discussed in the original papers. However, the review clearly suggests that self‐conscious emotions and related self‐appraisals, particularly negative ones, are relevant to many UK women's experiences of varied types of breastfeeding support. Though women may not always discuss these emotions explicitly in interviews, or experience them strongly, many women can find their mothering identity precarious and in need of active management when interacting with supporters. Careful emotion work is required to avoid feeling guilt, shame, embarrassment or even humiliation. As indicated in a recent review (Russell et al., 2021), those supporting breastfeeding are often entering an emotionally complex situation where mothers can feel embarrassment about breaching social norms, guilt about not meeting perceived moral obligations to breastfeed (or to fulfil duties incompatible with breastfeeding) and shame about perceived ‘failures’. Supportive interactions that do not shame or humiliate women further and that support positive emotion are vital.

The review focused on the United Kingdom. However, findings are likely to be transferable to contexts with similar breastfeeding support services and where cultural messages around infant feeding mean women approach breastfeeding support with a similarly conflicted view of themselves as breastfeeding mothers. This review supports Thomson et al.'s (2015) conclusion that such cultures can simultaneously shame mothers for breastfeeding and shame them for formula feeding or struggling with breastfeeding. Although there were no explicit references to shame in the reviewed papers, there were many references to feeling inadequate, deviant, exposed, excluded or a failure. This supports the contention that discussion of guilt or discomfort around infant feeding may sometimes mask unspoken experiences of shame (Leeming, 2018; Taylor & Wallace, 2012).

Although some women primarily approach breastfeeding support for help with practicalities, or require little support, the review demonstrates that others can receive far more from ‘breastfeeding’ interventions than help with successful breastfeeding. Positive validation can help to counter negative self‐beliefs and support women in developing a positive mothering identity that is resilient to shame, guilt and embarrassment. Positive relationships with others *can* also foster pride and a sense of achievement. However, although there was strong evidence of women internalising perceived inadequacies with infant feeding as failure, there were comparatively few examples of positive infant feeding experiences being internalised as a personal success.

Unfortunately, a number of women in the papers reviewed had experienced aspects of breastfeeding support as undermining and invalidating. Therefore, ‘support’ sometimes seemed risky and some women's apparent strategies for managing this risk—selective engagement and withdrawal, ‘illusions of compliance’ or externalisation of blame—can reduce the likelihood of receiving appropriate advice and of validating relationships with health care providers. Responses of self‐denigration were also apparent across several papers, which, in some situations, may increase the risk of mental health difficulties postnatally (Dunford & Granger, 2017).

### 4.1 Implications for health promotion and health care practice

Attempts to shift the moral and hence emotional landscape of infant feeding in the United Kingdom are already underway and have focused on ‘changing the conversation’:

It is time to stop laying the blame for the UK's low breastfeeding rates in the laps of individual women and instead acknowledge that this is a public health imperative for which government, policy makers, communities and families all share responsibility (UNICEF UK, 2016b).

This is one of the most useful ways in which public discourse around breastfeeding could change. It reduces blame, shame and guilt, while targeting key environmental influences on breastfeeding such as social discourse and availability of support and can further empower women by emphasising the protection of their *rights* to breastfeed (Trickey, 2016). Challenging the idea of fixed all‐or‐nothing breastfeeding ‘rules’, which create a perception of failure when these are broken, may also be helpful. Likewise, presenting a more nuanced understanding of breastfeeding as a ‘natural’ process may prevent breastfeeding being idealised and romanticised in a way that erases the possibility of difficulties and means women internalise challenges as personal deficiencies (Leeming, 2018). Instead, it seems useful to communicate expectation of an ‘adjustment period’ (Trickey & Newburn, 2014) to women and their families. The theme ‘precarious identity’ also points to the importance of working with families or community groups to shift devaluation of breastfeeding and challenge the idea that it might be self‐indulgent or exhibitionist. Some women seemed to be turning to formula milk to be ‘good’ mothers within their social networks, rather than because of an initial preference for this. However, the promotion of pride in breastfeeding may need careful thought. An overemphasis on pride in personal achievement raises the possibility of shame where there is perceived failure (Shepherd et al., 2017). Nonetheless, pride may be useful for asserting a collective identity as breastfeeding women (Mecinska, 2018), and it is worth unpacking the different meanings of ‘pride’, as some may be more helpful than others (Wubben et al., 2012).

There is increasing recognition of the importance of respectful conversations with mothers about infant feeding, which are empathic, person‐centred and non‐judgemental (e.g., Schmied et al., 2011; UNICEF UK, 2016a). This review extends understanding of why this validating approach is useful and fits with Gilbert's (2017) argument that compassion, kindness and acceptance can counter feelings of shame, embarrassment and humiliation. Validation not only boosts confidence but also reduces the risk of emotional responses that disrupt relationships between mothers and supporters. Recently developed peer support interventions are promising in that they explicitly incorporate a validating woman‐centred ethos and consideration of emotional responses, based on the Solihull Approach (Tan et al., 2017), motivational interviewing (Copeland et al., 2019) or an assets‐based approach (Ingram et al., 2020, published after present review). However, our review suggests that some environments, particularly inpatient postnatal settings, may facilitate invalidation rather than validation. Those supporting infant feeding need good emotional ‘antennae’ because, although self‐conscious emotions are common, they can go ‘underground’, particularly shame, and wreak unacknowledged havoc (Scheff, 2003), through the disengagement, subterfuge or hostility discussed above. Self‐conscious emotions are not clearly expressed via facial expressions (Robins & Schriber, 2009), and Gibson's (2015) analyses of social work suggest that shame and related feelings may be concealed in defensiveness, withdrawal, indirectness, hesitation and negative self‐evaluation. Those offering support may need to be alert to these cues. Although women sometimes referred to breastfeeding supporters as good listeners, none of the studies reviewed explicitly mentioned participants discussing self‐conscious emotions with supporters other than family/friends. However, this may have been unreported. Research on resilience to shame, probably the most toxic of the self‐conscious emotions, suggests that articulating shame may be key to tolerating it, creating opportunities for others to offer affirmation and alternative interpretations (Brown, 2006). Good emotional ‘antennae’ might prompt discussion of emotions and also guide maternity care workers' decisions about whether women are likely to engage comfortably with more exposed settings such as peer support groups.

### 4.2 Future research

Further research into the emotional impact of breastfeeding support interventions might help to shed light on the question raised previously as to why particular breastfeeding support interventions may be successful sometimes, but not others (e.g., Hoddinott et al., 2011). The current findings illustrate how interventions or support services that appear similar in design may have very different emotional effects for service users, depending on the relationships between those giving and receiving support (Leeming et al., 2017). It may be the case that researching *how* breastfeeding support is experienced by different women within different relationships and contexts is as important as researching what works, when ‘what’ is defined in broad brush terms as a packaged intervention. In‐depth ethnographic examination of how the cultural or organisational context shapes support relationships and behaviours might also enable better understanding of how some postnatal inpatient settings foster invalidating interactions whereas others foster validation. Gibson (2019) notes how organisational practices shape not just relationships but possibilities for shame and pride for the professionals working within them and their clients and how the experience of each party influences that of the other. The sense of guilt, failure, shame or pride that those supporting breastfeeding may feel in relation to organisational infant feeding targets has been outside the scope of this review. However, further exploration of the co‐construction of self‐conscious emotions between mothers, those supporting them and the organisations they work within may help to minimise devaluing interactions and ensure that breastfeeding interventions truly support breastfeeding mothers.

### 4.3 Evaluation of study

By beginning with a clear conceptual framework for our synthesis, we offer a new lens on breastfeeding interventions. This does mean that our approach draws attention to some issues rather than others. However, these issues may often be hidden and underresearched due to their taboo nature. This is the first review to explore the role of self‐conscious emotions in breastfeeding support. However, it should be seen as opening an area for further research rather than offering a detailed examination of emotion processes. We used a broad conceptualisation of self‐conscious emotions, focusing on accounts of self‐evaluation and concern about one's worth in the eyes of others, whether or not emotions were mentioned explicitly. Our aim was to include experiences where self‐conscious emotions might be on the periphery of consciousness but avoided through careful self‐management. This approach was important given the cultural preference for indirect references to some self‐conscious emotions, the assumption that unacknowledged self‐conscious emotions can still profoundly shape interactions (Scheff, 2003) and the fact that many of the papers reviewed had not intended to probe emotion processes in depth. However, this inclusive approach makes it difficult to distinguish between self‐evaluations that were accompanied by noticeable affect and those that were not. Further research, using methods that enable more in‐depth recounting of troubling and taboo emotions, will help us to understand more fully how self‐conscious emotions are *felt* by women in the context of receiving breastfeeding support, how this can shape their interactions with health care providers and how these interactions can be rethought to ensure they support mothers and protect breastfeeding.

## CONFLICT OF INTEREST

The authors declare that they have no conflicts of interest.

## ETHICS STATEMENT

Approval for this review was not required by the institutional ethics panel as the findings in the papers reviewed were already in the public domain and ethical approval had been obtained by the original researchers.

## CONTRIBUTIONS

DL and JM designed the study. JM and SH conducted the searches. DL, JM and SH coded and analysed the data. DL designed the original template and audited the final analysis. DL wrote the initial draft of the paper. DL, JM and SH reviewed and edited the paper and approved the final manuscript.

## Data Availability

Data sharing is not applicable to this article as no new data were created or analysed in this study.

## Footnotes

^1^
Quotations in standard font are statements by the original authors. Quotations in italics are from participants in the original study.
